# Altered Response to A(H1N1)pnd09 Vaccination in Pregnant Women: A Single Blinded Randomized Controlled Trial

**DOI:** 10.1371/journal.pone.0056700

**Published:** 2013-04-18

**Authors:** Anne Louise Bischoff, Nilofar Vahman Følsgaard, Charlotte Giwercman Carson, Jakob Stokholm, Louise Pedersen, Maria Holmberg, Amalie Bisgaard, Sune Birch, Theodore F. Tsai, Hans Bisgaard

**Affiliations:** 1 Copenhagen Prospective Studies on Asthma in Childhood (COPSAC), Health Sciences, University of Copenhagen, Copenhagen University Hospital, Gentofte, Denmark; 2 Novartis Vaccines, Cambridge, Massachusetts, United States of America; National Taiwan University Hospital, Taiwan

## Abstract

**Background:**

Pregnant women were suspected to be at particular risk when H1N1pnd09 influenza became pandemic in 2009. Our primary objective was to compare the immune responses conferred by MF59®-adjuvanted vaccine (Focetria®) in H1N1pnd09-naïve pregnant and non-pregnant women. The secondary aims were to compare influences of dose and adjuvant on the immune response.

**Methods:**

The study was nested in the Copenhagen Prospective Studies on Asthma in Childhood (COPSAC_2010_) pregnancy cohort in 2009-2010 and conducted as a single-blinded block-randomised [1∶1∶1] controlled clinical trial in pregnant women after gestational week 20: (1) 7.5 µg H1N1pnd09 antigen with MF59-adjuvant (Pa7.5 µg); (2) 3.75 µg antigen half MF59-adjuvanted (Pa3.75 µg); (3) 15 µg antigen unadjuvanted (P15 µg); and in non-pregnant women receiving (4) 7.5 µg antigen full adjuvanted (NPa7.5 µg). Blood samples were collected at baseline, 3 weeks, 3 and 10 months after vaccination, adverse events were recorded prospectively.

**Results:**

58 pregnant women were allocated to Pa7.5 µg and 149 non-pregnant women were recruited to NPa7.5 µg. The sero-conversion rate was significantly increased in non-pregnant (NPa7.5 µg) compared with pregnant (Pa7.5 µg) women (OR = 2.48 [1.03–5.95], p = 0.04) and geometric mean titers trended towards being higher, but this difference was not statistically significant (ratio 1.27 [0.85–1.93], p = 0.23). The significant titer increase rate showed no difference between pregnant (Pa7.5 µg) and non-pregnant (NPa7.5 µg) groups (OR = 0.49 [0.13–1.85], p = 0.29).

**Conclusion:**

Our study suggests the immune response to the 7.5 µg MF59-adjuvanted Focetria® *H1N1pnd09* vaccine in pregnant women may be diminished compared with non-pregnant women.

**Trial Registration:**

ClinicalTrials.gov NCT01012557.

## Introduction

Pregnant women experience increased influenza related morbidity and mortality during seasonal influenza epidemics,[1]–[3], and even graver outcomes during influenza pandemics [4], [5]. The WHO and many countries therefore prioritized pregnant women among those to receive the first available doses of H1N1pnd09 vaccine [6], [7]. Indeed, influenza hospital admission and mortality rates were higher for pregnant women than in the general population during the 2009 pandemic [8]–[10]; in addition, infected pregnant women experienced increased rates of stillbirth, perinatal and neonatal mortality than their non-infected counterparts [11]. The pathophysiology of increased influenza-related morbidity during pregnancy is not fully defined but an altered immune response to infection associated with immunological adaptations to pregnancy itself may contribute [12]. The immune response of pregnant women to influenza vaccine has not been well studied, as vaccination during pregnancy has not been recommended until recently [2], [3], [13], [14]. Although it has been widely held that responses to influenza vaccine in pregnant women and non-pregnant women are indistinguishable [15], [16], a recent study reported that antibody responses to seasonal influenza vaccination were lower in pregnant than in non-pregnant women [17]. Moreover, the reduced immune response of pregnant women to live-attenuated yellow fever vaccine suggests that the immune response of pregnant women to other antigens also may be impaired [18]. To our knowledge, no studies comparing immune responses to the H1N1pnd09 vaccine in pregnant and non-pregnant women have been reported.

We had the unique opportunity to study the *H1N1pnd09* vaccination of pregnant and non-pregnant women in our unselected, prospective, clinical pregnancy-cohort: the Copenhagen Prospective Study on Asthma in Childhood 2010 (COPSAC_2010_) recruited between Q1-2009 and Q4-2010. The timing of this enrolment and the pandemic provided for an “experiment of nature” in our population of subjects of whom half had completed pregnancy before the pandemic and the other half were pregnant while H1N1pnd09 virus was prevalent in the community.

We conducted a randomized controlled clinical trial primarily to compare immunogenicity of the *H1N1pnd09* vaccine in pregnant versus non-pregnant women; secondarily to study dose-related immune responses and adverse events to MF59-adjuvanted versus non-adjuvanted vaccine in pregnant recipients.

## Materials and Methods

This study is reported in accordance with the CONSORT guidelines [19]. The protocol (Protocol S1) for this trial and supporting CONSORT checklist (Checklist S1) are available as supporting information.

### Study design

This study was nested in the novel COPSAC_2010_ cohort; an on-going, unselected, prospective clinical pregnancy cohort study of 743 women recruited in Zealand, Denmark, during 2009–2010. The recruitment was previously described in detail [20].

The women participating in the COPSAC_2010_ cohort were invited to be enrolled in this phase IV randomized, participant-blinded study in 2009–2010. The pregnant women were recruited from gestational week 20 and women continuing in the birth cohort with their children were recruited up to 8 months after birth. Key exclusion criteria were; chronic endocrinological, nephrological or cardiac diseases; severe asthma; history of anaphylaxis or other serious vaccine reactions; or hypersensitivity to influenza viral proteins, any excipients, eggs (including ovalbumin), or chicken proteins.

Monovalent influenza A/California/2009 (*H1N1pnd09*) surface-antigen vaccine (Focetria, Novartis Vaccines and Diagnostics GmbH, Marburg, Germany) in both MF59-adjuvanted and non-adjuvanted forms was used, formulation of which is previously described in detail [21], [22]. The study consisted of four groups: Pregnant women: (1) 7.5 µg hemagglutinin (HA) with a full complement of MF59-adjuvant (as used in the licensed seasonal adjuvanted vaccine – containing 9.75 mg squalene, 1.175 mg polysorbate 80 and 1.175 mg sorbitan trioleate in a citrate buffer) (Pa7.5 µg); (2) 3.75 µg HA, with half the usual content of MF59 (Pa3.75 µg); (3) 15 µg HA, unadjuvanted vaccine (P15 µg); and (4) non-pregnant women receiving 7.5 µg HA with full MF59-adjuvanted vaccine (NPa7.5 µg). After recruitment, the pregnant women were randomized by a computer-generated permuted-block-randomization table, in blocks of 45 women with allocation rate 1∶1∶1. The Pa3.75 µg group was closed after four months due to low recruitment rate. The protocol was amended and a new computer-generated permuted-block-randomization table was made. The women were subsequently randomly assigned to the remaining two groups in 1∶1 ratio. The women were enrolled by research doctors.

The vaccines were portioned, in identical syringes, into two different individual dosages. One for the 7.5 µg full MF59-adjuvanted and 3.75 µg half MF59-adjuvanted dosages and one for the 15 µg unadjuvanted dose. The vaccine was administered as an intramuscular injection into the deltoid muscle. The women were observed for 30 minutes after the injection.

### Ethics Statement

The study was conducted in accordance with the guiding principles of the Declaration of Helsinki and approved by the Ethics Committee of Copenhagen (H-B-2008-093), the Danish Medicines Agency (EudraCT 2009-016877), and the Danish Data Protection Agency (2009-41-4031). The validity of the data was ensured by complying with Good Clinical Practice guidelines and quality-control procedures. (ClinicalTrials.gov number, NCT01012557). All participants gave their written informed consent prior to enrollment.

### Adverse events

The women visited the research clinic after three weeks for a structured clinical interview performed by the research doctors interviewing for specific local reactions (pain, erythema, swelling, and bruising), systemic symptoms (chills, malaise, headache, myalgia, nausea, and vomiting), fever, and use of analgesics. Symptoms and reactions were graded as: none; mild (did not interfere with daily activity); moderate (interfered with daily activity); and severe (prevented daily activity). At two further clinical visits, after three and 10 months, unsolicited events affecting mother or child were recorded. Any reaction that resulted in hospitalization or was life-threatening was considered as a serious adverse event.

### Serology

Serum was sampled prior to vaccination (baseline), 3 weeks, 3 months, and 10 months after vaccination. Serum plasma levels of antibodies were determined in twofold dilutions in a conventional hemagglutination-inhibition (HI) assay [23]. using turkey red blood cells [24]. Four hemagglutination units of a live viral preparation of A/CA/07/2009 (the strain contained in the pandemic vaccine), were used as the antigen. The coded samples were analyzed at Novartis Vaccines and Diagnostics, Marburg, Germany.

Sero-conversion was defined as a change from a pre-vaccination HI titer <10 to a post-vaccination HI titer ≥40; significant increase as fourfold or higher post-vaccination titer from a pre-vaccination HI titer ≥10, and sero-protection as the proportion of subjects achieving an HI titer of 40 or greater. See Table 1 for serology definitions.

**Table 1:** Definitions.

Sero-protection:		HI titer ≥40
**Sero-conversion:**	Pre-vaccination:	HI titer ≥40
	Post-vaccination:	HI titer ≥40
**Significant increase:**	Pre-vaccination:	HI titer ≥10
	Post-vaccination:	HI titer ≥4 x pre-vaccination titer

The HI antibody response-criteria for success in young adults as defined by the EMEA criteria [25] of successful response in young adults are: (1) >40% of subjects with sero-conversion or significant titer increase, (2) >70% of subjects achieving sero-protection; or a (3) Geometric Mean Ratio (GMR) between pre- and post-vaccination titers of >2.5.

### Statistics

The study power estimate was based on a minimal detectable Geometric Mean Titer (GMT) ratio of 1.5 between the Pa7.5 µg and the NPa7.5 µg groups, a coefficient of variation (CV) of 1.0, a ratio of 1:3 between number of pregnant and number of non-pregnant women, a power of 80%, and an two-sided alpha-level of 5%. This required us to recruit a total number of 180 women in the Pa7.5 µg and the NPa7.5 µg groups.

GMT were computed from the log_10_-transformed mean. GMT in the vaccination groups are compared in levels and trends over time using general estimating equations (GEE) analysis (repeated-measures analysis) with an independent working correlation structure. The motivation for using GEE analysis is to take advantage of the full data in order to report one overall conclusion on the difference in sero-conversion rates between the groups over time. Differences in GMT between groups are reported as ratios (R). Confidence intervals are reported in squared brackets. GMT at 3 weeks, 3 and 10 months are adjusted for baseline GMT, using baseline GMT as a covariate. Analysis on the Pa7.5 µg, Pa3.75 µg and P15µg groups were adjusted for gestational age at time of vaccination.

GMR are the ratio between baseline titer and titer at any time point.

Percentage of women sero-converted is calculated as the percentage of women with a pre-vaccination HI titer <10 who converted to a post-vaccination HI titer ≥40. Percentage of women with significant titer increase is calculated as the percentage of women with a pre-vaccination HI titer ≥10 who had a fourfold or higher post-vaccination titer. Percentage of women with sero-conversion or significant increase is calculated as percentage of the total number of women in the vaccination group.

Sero-protection, sero-conversion and significant titer increase in the vaccination groups are compared in levels and trends over time using general estimating equations (GEE) analysis (repeated-measures analysis) with an independent working correlation structure. Differences in sero-conversion and significant titer increases between groups are reported as longitudinal odds ratios (OR). Confidence intervals are reported in squared brackets. Due to large scatter in gestational age at time of vaccination in the pregnant women, analyses on the Pa7.5 µg, Pa3.75 µg and P15 µg groups were adjusted for gestational age at time of vaccination.

Local and systemic post-vaccination reactions are reported as percentages based on number of women and severity. We used Fisher's exact test to compare vaccine groups. Exact (Clopper-Pearson) confidence intervals are reported for all proportional endpoints in squared brackets.

A p-value <0.05 was considered as significant.

Analyses were done using SAS version 9.2 (SAS Institute, Cary, NC).

## Results

### Baseline characteristics

**Figure 1:**
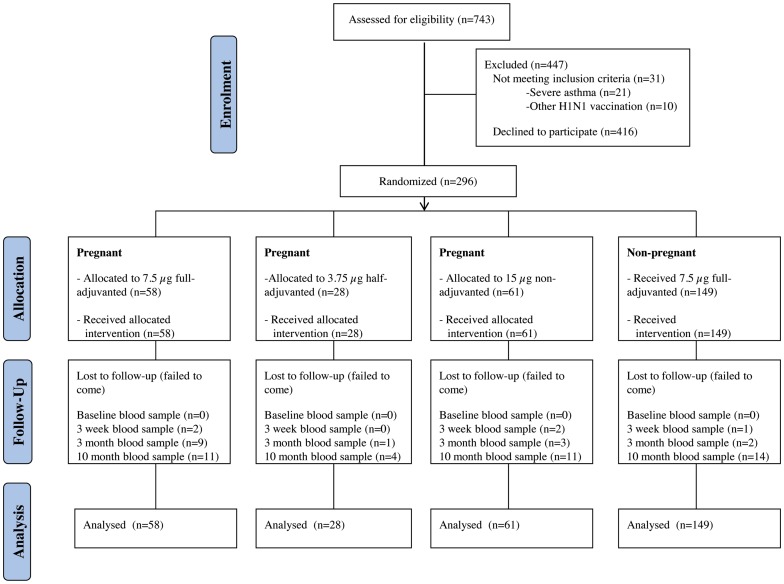
Study group flowchart.

296 women were included in this study from November 2009 to August 2010 of which 149 were non-pregnant (NPa7.5 µg). The 147 pregnant women were randomly assigned to the three groups Pa7.5 µg (58 women), Pa3.75 µg (28 women), and P15 µg (61 women). The Pa3.75 µg group was closed after four months due to a lower-than-expected recruitment rate to the study. There was a follow-up of 87% of the women 10 months after vaccination and the study was closed July 2011. Flow of subjects into the study-analysis is illustrated in the Figure 1, and the baseline characteristics of the study groups in Table 2.

**Table 2:** Baseline characteristic of the study subjects according to vaccine group.

	Pa7.5µg MF59- adjuvanted	Pa3.75µg MF59- adjuvanted	P15µg Not- adjuvanted	NPa7.5µg MF59- adjuvanted	P
N	58	28	61	149	
Age – year Median (Range)	32(19–40)	34(26–40)	31(20–41)	32(23–49)	0.12^A^
Gestational age at vaccination – weeks Median (Range)	25+0 (21+4 – 40+1)	29+0 (21+1 – 38+6)	24+6 (20+0 – 37+0)		*
Time from birth to vaccination – days Median (Range)				127 (6–244)	
**Income (pr. year) %(n)**					0.08^F^
<400.000 Dkkr	9(5)	4(1)	22(13)	13(19)	
400.000–1.000.000 Dkkr	82(45)	96(27)	73(43)	84(125)	
>1.000.000 Dkkr	9(5)	0	5(3)	3(5)	
**Previous births %(n)**					0.21^F^
Yes	48(28)	68(19)	64(39)	55(82)	
No	51(30)	32(9)	36(22)	45(67)	
Living with partner %(n)					0.69 F
Yes	100(58)	100(28)	98(60)	97(144)	
No	0	0	2(1)	3(5)	

A ANOVA.

F Fisher's exact test.

* The gestational age at time of vaccination is different in the Pa3.75 µg group compared with the Pa7.5 µg group (P = 0.01). There is no significant difference between Pa7.5 µg and P15 µg (P = 0.82). Wilcoxon Rank Sum Test (t approximation).

### Immunogenicity

We saw an overall successful sero-protective response from the H1N1pnd09 vaccination in more than 91% of pregnant and 99% of non-pregnant women as defined by one of the EMEA criteria [25]. The detailed immune responses are presented in Table 3.

**Table 3:** Antibody responses according to vaccine groups.

	Vaccine group			
	Pa7.5µg	Pa3.75µg	P15µg	NPa7.5µg
**Baseline**				
N	58	28	61	149
GMT †[95% CI]	10[8]–[13]	11[7]–[17]	9[7]–[12]	12[10]–[14]
Sero-protection‡ %[95% CI]	17[9]–[29]	17[6]–[37]	13[6]–[24]	13[8]–[19]
**3 Weeks**				
N	56	28	59	148
GMT *[95% CI]	345[244–487]	206[126–335]	202[145–283]	465[374–575]
GMR [95%CI]	33.2[22.5–49.2]	18.8[10.8–32.8]	21.7[14.8–31.8]	40.5[31.8–51.5]
Sero-protection‡ %[95% CI]	96[88–100]	89[72–98]	88[77–95]	99[95–100]
Sero-conversion %[95% CI]	95[82–99]	86[57–98]	83[67–93]	98[91–100]
Significant increase %[95% CI]	95[74–100]	93[66–100]	89[67–99]	86[76–94]
Sero-conversion or Significant increase %[95% CI]	95[85–99]	89[72–98]	85[73–93]	93[87–96]
**3 Months**				
N	49	27	58	147
GMT *[95% CI]	139[93–208]	87[51–150]	111[77–161]	207[164–261]
GMR [95%CI]	12.5[8.1–19.3]	8.0[4.4–14.4]	11.5[7.7–17.1]	18.0[14.0–23.1]
Sero-protection‡ %[95% CI]	82[68–91]	74[54–89]	76[63–86]	94[89–97]
Sero-conversion %[95% CI]	70[51–85]	64[35–87]	74[58–87]	91[83–96]
Significant increase %[95% CI]	84[60–97]	69[39–91]	63[38–84]	79[67–88]
Sero-conversion or Significant increase %[95% CI]	76[61–87]	67[46–83]	71[57–82]	86[79–91]
**10 Months**				
N	47	24	50	135
GMT *[95% CI]	75[48–118]	63[34–118]	85[55–130]	78[60–101]
GMR [95%CI]	6.8[4.2–10.8]	5.5[2.9–10.6]	8.4[5.3–13.2]	6.8[5.1–8.9]
Sero-protection‡ %[95% CI]	70[55–83]	67[45–84]	74[60–85]	75[66–82]
Sero-conversion %[95% CI]	59[39–76]	55[23–83]	70[51–84]	69[57–79]
Significant increase %[95% CI]	67[41–87]	54[25–81]	53[28–77]	59[46–71]
Sero-conversion or Significant increase %[95% CI]	62[46–75]	54[33–74]	64[49–77]	64[56–72]

P: Pregnant.

NP: Non-pregnant.

GMT: Geometric mean titer.

GMR: Geometric mean ratio.

CI: Confidence interval.

Sero-protection: Titer ≥40.

Sero-conversion: Pre titer<10, Post titer ≥40.

Significant increase: Pre titer ≥10, Post titer 4 fold Pre titer.

Sero-conversion or significant increase: Percentage of total number of women in each vaccine group.

† At day 0 GMT were the same in all groups (ANOVA, p = 0.51).

‡ The number of women with sero-protection did not differ in any of the groups compared over time (GEE).

* GMT at 3 weeks, 3 months and 10 months were adjusted for baseline titer.

**Figure 2:**
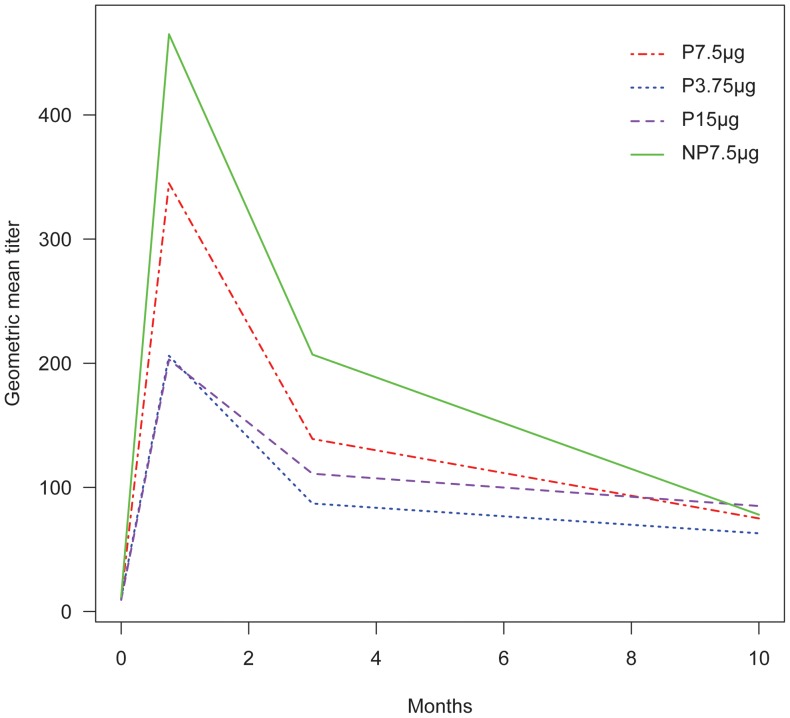
Evolution of geometric mean titers in the four vaccine groups over time. The trends over time for geometric mean titers in the vaccine groups were not significantly different using general estimating equations.

The CV for GMT was 1.8. Subjects with baseline titers >40 were found in all groups. Therefore, GMT at 3 weeks, 3 months and 10 months were adjusted for baseline titer (Figure 2 and Table 3).

#### Time-trend

**Figure 3:**
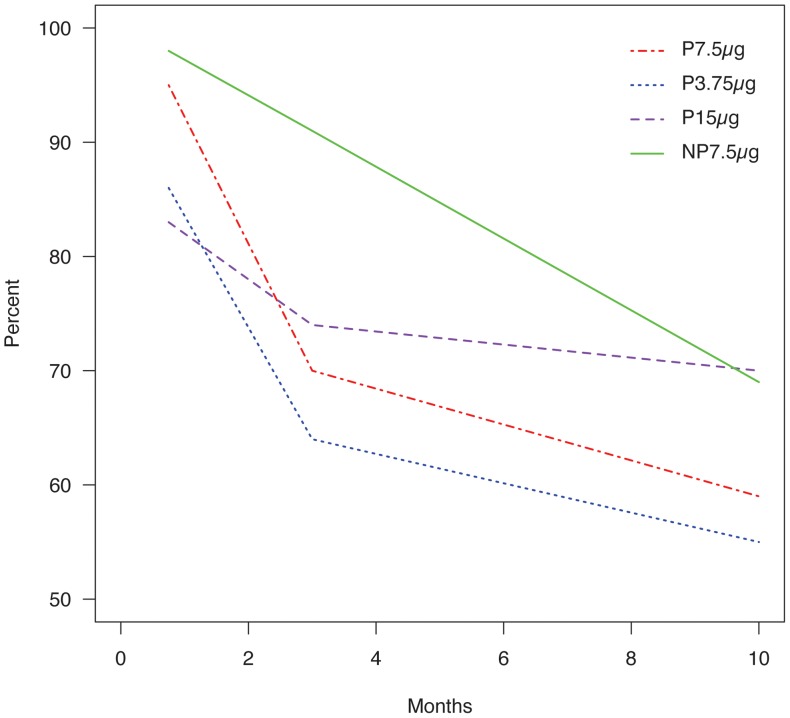
Evolution of sero-conversion rate (as percentage) in women in the four vaccine groups over time. The trends over time for the number of women sero-converted were not significantly different. The number of non-pregnant women (NPa7.5 µg) who sero-converted was 2.48-fold higher than among pregnant (Pa7.5 µg) women using general estimating equations.

The interactions with time of the vaccine groups were not significant; i.e. the trends over time for the number of women sero-converted, and for the women with significant titer increase were not significantly different when comparing pregnant (Pa7.5 µg) *versus* non-pregnant (NPa7.5 µg) women; comparing Pa3.75µg group *versus* Pa7.5 µg group ; and comparing P15 µg group *versus* Pa7.5 µg group (Figure 2–3).

#### Pregnant versus non-pregnant women

GMT numerical values (Table 3) suggested a lower response in pregnant women (Pa7.5 µg) compared with non-pregnant women (NPa7.5 µg) but this was not significant (R of non-pregnant vs pregnant women: 1.27 [0.85–1.93], p = 0.23) (Figure 2, Table 3). The number of non-pregnant women (NPa7.5µg) who sero-converted was 2.48-fold higher than among pregnant (Pa7.5 µg) women (OR = 2.48 [1.03–5.95], p = 0.04) (Figure 3, Table 3). The number of women with significant titer increase from baseline was not significantly different (OR = 0.49 [0.13-1.85], p = 0.29) (Table 3).

#### Dose-related response in pregnant woman

There was no significant difference between the Pa3.75µg group and the Pa7.5 µg group with respect to GMT (R = 0.63 [0.33-1.24], p = 0.18) (Figure 2, Table 3); sero-conversion (OR = 0.65 [0.18-2.39], p = 0.52) (Figure 3, Table 3) or the number of women with significant titer increase in the two groups (OR = 0.41 [0.10–1.77], p = 0.23) (Table 3). Importantly, the confidence limits are very wide corresponding to the low number of participants in the P3.75 µg group.

#### Response to non-adjuvant, double-dose vaccine in pregnant women

There was no significant difference between the P15µg group and the Pa7.5µg group with respect to GMT (R = 0.83 [0.48–1.42], p = 0.49) (Figure 2, Table 3); sero-conversion (OR = 1.11 [0.42–2.91], p = 0.83) (Figure 3, Table 3) or the number of women with significant titer increase in the two groups (OR = 0.33 [0.07–1.55], p = 0.16) (Table 3).

### Safety analysis

Local and systemic reactions in the first 3 weeks following vaccination are shown in Table 4 and 5. Overall, 72% of women reported adverse reactions, 23% reported no adverse reactions, and 4% had missing information on the adverse reactions. 64% experienced local reactions after vaccination. The women in the P15 µg group experienced significantly fewer local reactions than in the Pa7.5 µg and NPa7.5 µg groups with respect to pain (P<0.000001), erythema (P = 0.03), and swelling (P = 0.008) (Table 4). 26% of the women reported systemic reactions, with malaise as the most common symptom in 20% of the women. There were no significant differences between reported systemic reactions in the four study groups (Table 5). There were no serious adverse events in this study.

**Table 4:** Solicited local reactions the first 3 weeks following vaccination.

		Pa7.5µg MF59-adjuvanted	Pa3.75µg MF59-adjuvanted	P15µg Not-adjuvanted	NPa7.5µg MF59-adjuvanted
N		58	28	61	149
Missing data %(n)		5(3)	7(2)	8(5)	2(3)
**Local reaction**		*Percentage [95% confidence interval]*			
Pain*	None	18[9]–[30]	27[12]–[48]	82[70–91]*	24[17]–[32]
	Mild	56[42–70]	69[48–86]	16[8]–[28]	61[52–69]
	Moderate	22[12]–[35]	4[0–20]	2[0–10]	14[9]–[21]
	Severe	4[0–13]	0[0–13]	0[0–6]	1[0–4]
Erythema**	None	89[78–96]	96[80–100]	98[90–100]**	89[83–94]
	Mild	7[2]–[18]	4[0–20]	0[0–6]	10[6]–[16]
	Moderate	4[0–13]	0[0–13]	2[0–10]	1[0–4]
	Severe	0[0–6]	0[0–13]	0[0–6]	0[0–2]
Swelling***	None	85[73–94]	81[61–93]	98[90–100]***	87[81–92]
	Mild	15[7]–[27]	19[7]–[39]	0[0–6]	11[6]–[17]
	Moderate	0[0–6]	0[0–13]	2[0–10]	2[0–6]
	Severe	0[0–6]	0[0–13]	0[0–6]	0[0–2]
Bruising	None	93[83–98]	96[80–100]	96[88–100]	95[90–99]
	Mild	5[1]–[15]	0[0–13]	2[0–10]	4[2]–[9]
	Moderate	2[0–10]	4[0–20]	2[0–10]	1[0–4]
	Severe	0[0–6]	0[0–13]	0[0–6]	0[0–2]

Mild: Not interfering with daily activity.

Moderate: Interfering with daily activity.

Severe: Preventing in engaging in daily activity.

* The number of women who experienced pain as an adverse event were significantly less in the group vaccinated with the 15 µg non-adjuvanted vaccine, than in the other groups (P<0.000001, Fisher's exact test).

** The number of women who experienced erythema as an adverse event were significantly less in the group vaccinated with the 15 µg non-adjuvanted vaccine, than in the other groups (P = 0.03, Fisher's exact test).

*** The number of women who experienced pain as an adverse event were significantly less in the group vaccinated with the 15 µg non-adjuvanted vaccine, than in the other groups (P = 0.008, Fisher's exact test).

**Table 5:** Solicited systemic reactions the first 3 weeks following vaccination.

		Pa7.5µg MF59-adjuvanted	Pa3.75µg MF59-adjuvanted	P15µg Not-adjuvanted	NPa7.5µg MF59-adjuvanted
N		58	28	61	149
Missing data %(n)		5(3)	7(2)	8(5)	2(3)
**Systemic reaction**	*Percentage [95% confidence interval]*				
Chills	None	95[85–99]	100[87–100]	93[83–98]	91[85–95]
	Mild	5[1]–[15]	0[0–13]	4[0–12]	8[4]–[14]
	Moderate	0[0–6]	0[0–13]	2[0–10]	0[0–2]
	Severe	0[0–6]	0[0–13]	2[0–10]	1[0–4]
Malaise	None	78[65–88]	92[75–99]	86[74–94]	73[66–80]
	Mild	16[8]–[29]	8[1]–[25]	9[3]–[20]	19[13]–[26]
	Moderate	5[1]–[15]	0[0–13]	4[0–12]	6[3]–[11]
	Severe	0[0–6]	0[0–13]	2[0–10]	1[0–4]
Headache	None	89[78–96]	96[80–100]	89[78–96]	88[81–93]
	Mild	9[3]–[20]	4[0–20]	4[0–12]	7[4]–[13]
	Moderate	2[0–10]	0[0–13]	4[0–12]	3[1]–[7]
	Severe	0[0–6]	0[0–13]	4[0–12]	2[0–6]
Myalgia	None	91[80–97]	100[87–100]	96[88–100]	88[81–93]
	Mild	9[3]–[20]	0[0–13]	2[0–10]	10[5]–[15]
	Moderate	0[0–6]	0[0–13]	0[0–6]	2[0–6]
	Severe	0[0–6]	0[0–13]	2[0–10]	1[0–4]
Nausea	None	100[94–100]	100[87–100]	96[88–100]	93[88–97]
	Mild	0[0–6]	0[0–13]	0[0–6]	5[2]–[10]
	Moderate	0[0–6]	0[0–13]	4[0–12]	1[0–4]
	Severe	0[0–6]	0[0–13]	0[0–6]	1[0–4]
Vomiting	None	100[94–100]	100[87–100]	98[90–100]	99[95–100]
	Mild	0[0–6]	0[0–13]	2[0–10]	0[0–2]
	Moderate	0[0–6]	0[0–13]	0[0–6]	1[0–4]
	Severe	0[0–6]	0[0–13]	0[0–6]	1[0–4]
Fever	None	98[90–100]	96[80–100]	93[83–98]	95[90–98]
	>38° C	2[0–10]	4[0–20]	7[2]–[17]	5[2]–[10]
Analgesics	None	95[85–99]	96[80–100]	95[85–99]	90[85–95]
	Yes	5[1]–[15]	4[0–20]	5[1]–[15]	10[5]–[15]

Mild: Not interfering with daily activity.

Moderate: Interfering with daily activity.

Severe: Preventing in engaging in daily activity.

## Discussion

### Principal Findings

The sero-conversion OR was 2.48-fold in non-pregnant women (NPa7.5 µg) compared with pregnant women (Pa7.5 µg) after receiving the same standard H1N1pnd09 vaccine. Likewise GMT was nominally higher in the non-pregnant than in the pregnant women.

As young adults were at high risk for illness during the pandemic, it was of interest that even after 10 months, 70% −74% of pregnant (Pa7.5 µg and P15 µg) and 75% non-pregnant women (NPa7.5 µg) were protected against H1N1 according to the EMEA criteria with a HI titer of 40 or greater.

Women receiving the non-adjuvanted vaccine had significantly fewer local reactions but similar rates of systemic reactions as women receiving the adjuvanted vaccine. There were no reports of serious adverse events in any of the groups.

### Strengths and Limitations of the Study

It is a major strength to this study that it is nested in the on-going well established COPSAC_2010_ cohort study, which ensured a high follow-up rate and close observations in a clinical research centre.

The non-pregnant subjects were post-partum women drawn from the parent study and had delivered within 8 months prior to vaccination. As immune changes associated with pregnancy may be carried over transiently to the post-partum period, this would have had the effect of minimizing differences between pregnant and non-pregnant subjects, meaning that our observations were conservative.

It is a limitation of this study, that the recruitment was hampered by two main factors; primarily the Danish Health Authorities were ambiguous in their recommendations for vaccination of pregnant women changing their recommendations during the pandemic. This ambivalence caused the women to doubt the necessity and safety of the offered vaccination. Secondly the clinical symptoms of the pandemic proved less serious than expected. Therefore, failing to recruit at the scheduled rate, we chose to close the Pa3.75 µg treatment arm halfway into the recruitment period. The low number of participants in this study arm limits the power of the conclusion on this particular treatment.

As a consequence of lower than expected recruitment the power to detect differences between study arms was lower than planned.

The response to the unadjuvanted vaccine containing the usual 15 µg of antigen in the pregnant women can only be compared to the adjuvanted vaccine containing 7.5 µg of antigen in the non-pregnant women, since this study did not include a non-pregnant 15 µg dose unadjuvanted group. Hence we could not report on the adequacy of the usual unadjuvanted 15 µg antigen dose in pregnancy. It is a limitation of the study that it was not observer blind; but the extensive interviews for adverse events were part of the comprehensive clinic interviews in the main study with a focus on asthma, eczema and allergy and were integrated within the framework of the on-going COPSAC_2010_ study.

The serum plasma levels of antibodies were determined by only the HI assay. Using a second method e.g. the microneutralization assay [26], to determine the serum plasma levels of antibodies and comparing these values would have strengthened our results.

Finally, it is a limitation to our study (though inevitable), that the baseline GMT was raised in certain individuals in all four groups, indicating that some individuals in the population were already protected against the novel influenza H1N1pnd09. This could be due to the cross-reactivity from other influenza viruses [27], [28]. An earlier study on the Focetria vaccine showed that the presence of a high baseline titer did not have any influence on the rate of sero-conversion or titer increase [29].

### Interpretation

The increased risk for severe influenza in pregnant women was demonstrated again in the 2009 pandemic, underscoring the importance of protecting this vulnerable group by vaccination and with antiviral therapy [8]–[10]. Therefore the public health and medical imperative to protect pregnant women from influenza is clear and routine vaccination is increasingly recommended.

Vaccinating pregnant women against influenza may have a secondary benefit by passively protecting the parturient woman's infant,[30]–[33], and higher antibody titers achieved in pregnancy may increase the efficacy or duration of that transmitted immunity [34]–[36]. Infants under 6 months of age have high rates of serious influenza-related disease leading to hospitalizations while no influenza vaccine is licensed for children below 6 months old; therefore, routine vaccination of pregnant women has been proposed as a means to protect them indirectly [2], [33]–[38]. An additional unexpected benefit of vaccinating pregnant women during the pandemic was an improved outcome of pregnancy, including a ∼30% reduction in premature births, and reduced risks for fetal death and delivering small for gestational age neonates [39], [40].

The increased risk for severe influenza in pregnant women is not fully understood. Physiological changes associated with pregnancy play a role in increased severity of influenza. Immunity against secondary infections is also potentially important. At least one third of the deaths due to the pandemic were complicated by secondary bacterial infection [41]. While overall IgG levels normally decline in pregnancy, that depression has not, to our knowledge, been shown to lead to a reduction of influenza HI antibody titers or to contribute to the increased severity of influenza in pregnancy [42]. While antibodies are the principal means of protection against acquiring influenza, cytotoxic T cells do contribute to controlling the severity of infection by clearing viral infected cells. The immune response to vaccination in pregnant and non-pregnant women has been presumed to be similar, but supporting evidence is scarce [15], [16]. Immunological adaptations to pregnancy lead principally to a relative reduction of Th1 responses while protection against influenza in young adults is provided by antibodies (Th2) – conventionally measured by HI [43]–[45]. We showed that responses to vaccination in pregnant women may be slightly compromised compared with non-pregnant women, as demonstrated by a 2.48-lower rate of sero-conversion and lower GMT responses, compared with non-pregnant women receiving the identical MF59-adjuvanted vaccine containing 7.5 µg of HA. This observation is consistent with a report that pregnant women responded less well to unadjuvanted seasonal trivalent influenza vaccine, compared to non-pregnant control women [17]. It is unclear if this difference has any clinical consequence. Serological correlates of protection, established in human challenge studies and in field trials, have shown that, in young adults, an HI titer of ∼40 correlates with protection against acquiring illness [46], [47]. Given the potential for severe outcomes of pandemic influenza in pregnant women,[12], [45], [48], our data suggest the need for further comparisons of the responses in pregnant women to influenza viral strains with a pandemic potential with a view to confirm the effectiveness of the unadjuvanted 15 µg dose and potential benefits of higher doses to obtain sufficient protection of the pregnant woman herself and her infant [33], [49].

The response of pregnant women to unadjuvanted vaccine (containing 15 µg of HA) seemed adequate, though we observed numerically higher GMTs in response to adjuvanted vaccine containing one half the antigen, and equal responses to adjuvanted vaccine containing one fourth the antigen (3.75 µg). This degree of antigen-sparing from adjuvants has been observed consistently in previous studies in children, young and older adults [21], [22], [28], and has global public health implications because of the inadequate world supply of influenza vaccine [50]. Also consistent with previous experience, the adjuvanted vaccine was more locally reactogenic but did not increase systemic adverse reactions, including fever [29], [51]. This is an important feature for a vaccine to be administered in pregnancy, as fever may be teratogenic [52]–[54]. We observed no serious adverse events related to any of the vaccines.

## Conclusion

We find a significantly lower number of pregnant women who sero-converted than among non-pregnant women. Our study suggests a trend towards a reduced immune response to the 7.5 µg MF59 adjuvanted Focetria *H1N1pnd09* vaccine in pregnant compared with non-pregnant women. The adjuvanted vaccine was significantly more locally reactogenic but provided a similar and possibly higher immune response compared to non-adjuvanted, 15 µg *H1N1pnd09* vaccine in pregnant women.

## Supporting Information

Checklist S1 **CONSORT Checklist.**
(DOC)

Protocol S1 **Trial Protocol.**
(DOC)
