# Supplementary material for: Altered Response to A(H1N1)pnd09 Vaccination in Pregnant Women: A Single Blinded Randomized Controlled Trial
Source: PLoS One. 2013 Apr 18;8(4):e56700. doi: 10.1371/journal.pone.0056700 (PMC3630160; doi:10.1371/journal.pone.0056700)
Supplement: Protocol S1 — Trial Protocol. (DOC) [file pone.0056700.s002.doc]

| Copenhagen Studies on Asthma in Childhood (COPSAC) |
| --- |
| H1N1v Vaccination of Pregnant Women |
| *A Longitudinal Cohort Study Characterizing Influenza A-H1N1v Vaccination in Pregnant Women* |
| | Principal Investigator  Anne Louise Bischoff, MD, PhD Head of the Copenhagen Studies on Childhood Asthma (COPSAC) Translational Research Center Health Sciences, University of Copenhagen;  Copenhagen University Hospital, Gentofte; Copenhagen, Denmark. | | --- | |

Contents

[1 Summary 3](#__RefHeading___Toc242601129)

[2 Background 3](#__RefHeading___Toc242601130)

[2.1 Pregnant Women and Infants are at Risk during the Pandemic *H1N1v* 3](#__RefHeading___Toc242601131)

[2.2 Vaccination policies 4](#__RefHeading___Toc242601132)

[2.3 The current enrollment of a birth cohort provides a unique opportunity to study *H1N1v* in pregnant women 5](#__RefHeading___Toc242601133)

[2.4 Study Impact on Health Policy 5](#__RefHeading___Toc242601134)

[3 Research Objectives 5](#__RefHeading___Toc242601135)

[4 Study Design 6](#__RefHeading___Toc242601136)

[4.2 Sample Size and Power Estimate 7](#__RefHeading___Toc242601137)

[4.3 Recruitment strategy 7](#__RefHeading___Toc242601138)

[4.4 Participant Selection 7](#__RefHeading___Toc242601139)

[4.4.2 Inclusion criteria 7](#__RefHeading___Toc242601140)

[4.4.2 Exclusion criteria 7](#__RefHeading___Toc242601141)

[4.5 Study Withdrawal 8](#__RefHeading___Toc242601142)

[4.6 Recruitment Bias 8](#__RefHeading___Toc242601143)

[4.7 Immunogenicity End-Points 8](#__RefHeading___Toc242601144)

[4.8 Safety Endpoints 9](#__RefHeading___Toc242601145)

[5 Clinical Study Plan 9](#__RefHeading___Toc242601146)

[5.1 Clinical Study Plan, Pregnant Mothers 9](#__RefHeading___Toc242601147)

[6 Work Packages 9](#__RefHeading___Toc242601148)

[WP-1 Cohort Infrastructure 9](#__RefHeading___Toc242601149)

[WP-1.1 Recruitment of 800 pregnant women during 2^nd^ trimester 9](#__RefHeading___Toc242601150)

[WP-1.2 Recruitment of newborns into the ABC birth cohort 10](#__RefHeading___Toc242601151)

[WP-1.3 Database for on-line data capture 10](#__RefHeading___Toc242601152)

[WP-1.4 Data monitoring 10](#__RefHeading___Toc242601153)

[WP-2 Clinical surveillance 10](#__RefHeading___Toc242601154)

[WP-2.1 Scheduled visits 10](#__RefHeading___Toc242601155)

[WP-2.4 SAE reporting 10](#__RefHeading___Toc242601156)

[WP-3 H1N1v analyses 11](#__RefHeading___Toc242601157)

[WP-4 Vaccine Supply, Storage, Tracking and Labeling 11](#__RefHeading___Toc242601158)

[WP-5 Processing, Labeling and Storage of Serum Samples for Serology 12](#__RefHeading___Toc242601159)

[7 Data Management 12](#__RefHeading___Toc242601160)

[7.1 Data Collection 12](#__RefHeading___Toc242601161)

[7.2 Record Retention 13](#__RefHeading___Toc242601162)

[7.3 Data Monitoring 13](#__RefHeading___Toc242601163)

[7.4 Data Analysis Plan 13](#__RefHeading___Toc242601164)

[7.5 Data Reporting 13](#__RefHeading___Toc242601165)

[7.6 Data Sharing 13](#__RefHeading___Toc242601166)

[8 Legal and Ethical Aspects 13](#__RefHeading___Toc242601167)

[9 Study Impact 14](#__RefHeading___Toc242601168)

[9.1 Innovative value 14](#__RefHeading___Toc242601169)

[9.2 Publication and promotional strategy 14](#__RefHeading___Toc242601170)

[10 Feasibility and Project Management 15](#__RefHeading___Toc242601171)

[10.1 Research Management: 15](#__RefHeading___Toc242601172)

[10.2 Governance 15](#__RefHeading___Toc242601173)

[11 Co-Funding 15](#__RefHeading___Toc242601174)

[13 References 16](#__RefHeading___Toc242601175)

# 1 Summary

Pregnant women are at particular risk during the imminent *H1N1v* influenza pandemic. The new *H1N1v* virus requires urgent political and medical decisions on vaccination strategies in order to minimize severe disease and death from this pandemic. However, there is a lack of evidence to build such decisions upon. A vaccine will be provided in the fourth quarter of 2009, but there is little knowledge on the immunogenicity. Particularly its clinical effectiveness and duration of immunity in pregnant women and their newborn infants is unknown. Therefore, it will be important to study the optimal vaccination regimens with respect to dosing and use of adjuvant to decide future health policies on vaccination of pregnant women.

We have a unique possibility to study these aspects of *H1N1v* infection in pregnant women in our ongoing unselected, prospective, birth-cohort study recruiting 800 pregnant mothers between Q1-2009 and Q4-2010. Pregnant women from East-Denmark are being enrolled during the 2^nd^ trimester and their infant will undergo a close clinical follow-up. The *H1N1v* pandemic is expected to reach Denmark Q4-2009. The timing of this enrollment and the imminent pandemic allows for an “experiment of nature” whereby the first half of the mothers completes pregnancy before the *H1N1v* pandemic. The other half of this cohort will be pregnant while *H1N1v* is prevalent in the community and will require *H1N1v* vaccination.

The aim of this randomized, controlled, trial is to compare and evaluate the dose-related immune protection conferred by vaccine and adjuvant (Novartis vaccine *Focteria*) in pregnant women and non-pregnant women. In addition the protocol will assess the passive immunity conferred to the newborn from these vaccine regimes.

The study will provide evidence-based guidance for health policies on vaccination for the population of pregnant women during future *H1N1v* pandemics.

# 2 Background

## 2.1 Pregnant Women and Infants are at Risk during the Pandemic *H1N1v*

A new influenza A virus, *H1N1v*, is currently spreading throughout the world in the first pandemic of influenza in the last 40 years. It is expected to become epidemic in Denmark within the next few months.

Pregnant women are at particular risk from this novel pandemic. This is illustrated by a large overrepresentation of pregnant women among hospitalized individuals infected with *H1N1v* in countries where the epidemic has already taken effect. As an example, the first 15 confirmed *H1N1v* infections of pregnant women in the United States resulted in 3 hospitalizations of which one died <http://www.cdc.gov/mmwr/preview/mmwrhtml/mm5818a3.htm>. In total the CDC is currently reporting that approx 100 women has hospitalized in ICU and 28 have died from H1N1. In a subsequent analysis, pregnant women were reported to have a fourfold higher risk for hospitalization than cases in the general population.(1) The case-fatality ratio among the pregnant women was13% compared to an overall case fatality rate of 0.8%. In addition, an excess of influenza-associated deaths among pregnant women was reported during the pandemics of 1918–1919 and 1957–1958. <http://www2a.cdc.gov/HAN/ArchiveSys/ViewMsgV.asp?AlertNum=00285>. It is unclear why pregnant women are at higher risk for influenza-related complications than non-pregnant women of the same age but a recent observation suggests that a difference in the subclass of IgG antibodies that are elaborated during pregnancy could play a role.

Infants are also at high-risk. The virus seriously impacted infants in the recent epidemic in New Zealand,(2) presumably due to the fact that the older population has been infected with influenza strains in past years, which share antigen epitopes with this novel influenza A (*H1N1v*) viral strains.(3;4) The risk for severe complications is highest among children aged <1 years with a 5-7 fold increased hospitalization rate. (5-7). In a pilot study infants were protected through vaccination of the pregnant mother conferring passive immunity to the newborn.(8) However, this has not been documented in larger studies and needs investigations. This strategy of indirect protection is important because no influenza vaccine is licensed for infants under six months of age.

The new *H1N1v* virus requires urgent political and medical decisions on vaccination strategies in order to minimize severe disease and death from this pandemic in pregnant women and their newborn infants. However, there is a lack of evidence to build such decisions upon as reflected in grossly different approaches in different countries, particularly with respect to vaccination of two of the most susceptible groups; the pregnant women and their infants. Studies are needed to provide evidence from pregnant women on the vaccine immunogenicity, clinical effectiveness, optimal vaccine dose and its duration of protection in mother and child and the need for vaccine adjuvant.

## 2.2 Vaccination policies

Vaccine will be available in the fourth quarter of 2009. The immunogenicity and clinical effectiveness of this new vaccine is uncertain and without evidence from phase III trials. In preliminary reports of studies in adults, the monovalent influenza A 2009 (*H1N1v*) vaccine was immunogenic, with mild-to-moderate vaccine-associated reactions.(9;10) It is unknown how effective and lasting this immunity is and the optimal dosing regimens have not been studied.

Deciding the vaccination strategy in pregnancy is challenging because there is little knowledge about the immunity to H1N1v infection in pregnant women. Optimal vaccine strategy in pregnant women is urgent because this group is prone to experiencing severe symptoms to H1N1v infection as a result of changes in the immune system during pregnancy. Efficiency of vaccine immunization of the pregnant women must be studied to provide evidence-based patient management and aid policy decisions in relation to the emerging and future pandemics.

Several vaccines against H1N1v are emerging as a result of the eminent pandemic. The MF59 adjuvant is a metabolizable oil squalene with two surfactants, polyoxyethylene sorbitan monooleate and sorbitan trioleate, oil-in-water emulsion. MF59 is the adjuvant used in Fluad administered to elderly people not capable of mounting a sufficient immune response following immunization, and has been approved for use in Europe for over a decade. The Focetria vaccine (produced by Novartis) against H1N1v, containing the MF59 adjuvant, is highly immunogenic and confers a high degree of protection(10). Accordingly it is possible that this particular vaccine, or one containing no adjuvant, is capable of providing sufficient protective immunity against H1N1v infection. The proposed study offers a unique possibility to examine the effectiveness of this vaccine on pregnant women and children born to vaccinated women. It also offers a unique possibility to gain unprecedented insight into duration of vaccine protection, tolerance and safety of the Focetria vaccine as well as evaluation of the immune/vaccination response in pregnant women compared to non-pregnant women and the passive immunity conferred to the newborn child.

The proposed project will focus on elucidating vaccine efficiency during the 2nd and 3d trimester, as there is little documented experience with adjuvanted vaccines used in 1st trimester of pregnancy.

*Denmark:*

The Health Authorities provides vaccines corresponding to approx 1/3 of the population, aiming to prioritize certain risk-groups and personnel with key-functions in the society. Very recently the recommendation changed and now pregnant women and infants are no longer defined as high-risk population that will be offered vaccination.

*USA:*

CDC recommends that all pregnant women are vaccinated against influenza A (*H1N1v*) using the 2009 monovalent vaccine. This recommendation has been issued because pregnant women are at higher risk of complications and because the vaccine can potentially provide protection to infants who cannot be vaccinated. In addition, CDC recommend pregnant women as the highest prioritized group to receive vaccination if a shortage of vaccine is experienced.

## 2.3 The current enrollment of a birth cohort provides a unique opportunity to study *H1N1v* in pregnant women

Our ongoing longitudinal clinical birth cohort study is currently recruiting 800 pregnant mothers since Q1-2009 continuing into Q4-2010. This coincides with the novel *H1N1v* pandemic expected to reach our region in a few months, i.e. separating the cohort in two half’s. The first half of the pregnant women will give birth before infection and vaccine is prevalent in the community, while the second half will be pregnant after the *H1N1v* has become epidemic in this region.

The main focus of the birth cohort study is monitoring and objective assessment of respiratory infections and symptoms during early life with the aim to understand the origins of asthma. The research center conducting this study is highly experienced in handling the ethical and practical challenges associated with such intensive clinical studies through an earlier and similar birth cohort study.(11-17) This assures the feasibility and quality of the project.

This present protocol is nested into the main birth cohort study and is only possible because the logistics of the birth cohort study is already fully established and the first 300 of the targeted 800 have been enrolled before the imminent *H1N1v* pandemic. Core support for this study is already provided and the current protocol is piggy-bagging on the comprehensive study set-up.

## 2.4 Study Impact on Health Policy

This protocol aims to save lives and reduce severe symptoms and hospitalization of pregnant women and their infants from future *H1N1v* pandemic. We aim to improve the evidence base for an optimal vaccination strategy against *H1N1v* infection in pregnant women. This program will provide timely evidence-based guidance for health policies on vaccination for the population of pregnant women during future *H1N1v* pandemics, it will help designing future studies and will be valuable for the handling of future epidemics with new viruses.

# 3 Research Objectives

It is the overriding objective to study the optimal vaccination strategy in 2^nd^ - 3^rd^ trimester pregnant women with the objectives to:

- Assess whether the adjuvanted vaccine offers a meaningful benefit in pregnant women in terms of immune response over the non-adjuvanted vaccine, and if so, whether a low dose adjuvanted vaccine is sufficient.
- Assess the persistence of immune response over a 15 month time period after one single vaccination of the pregnant mother.
- Assess whether the immune response to H1N1 in pregnant women is different from that of non-pregnant women of similar age.
- Gain insight on safety of the Novartis egg-based MF59 adjuvanted H1N1 vaccine Focetria in pregnant women and their babies.
- Assess the maternally transferred specific antibodies against *H1N1v* in the newborn.

# 4 Study Design

This is a randomized, single-blind study.

We will assess the relative protection from two vaccine regimens comparing full dose adjuvanted vs half dose adjuvanted vs non-adjuvanted vaccine in >20 week pregnant women and compare these regimens to the efficiency of full-dose adjuvanted vaccine in non-pregnant women.

Study subjects will be randomized to one of four groups, each receiving one dose of three *H1N1v* vaccines (egg-based Novartis vaccine Focteria):

- Group A: Pregnant women (>20 weeks), 7.5 mcg *H1N1v* full MF59 adjuvant
- Group B: Pregnant women (>20 weeks), 3.75 mcg *H1N1v* half MF59 adjuvant
- Group C: Pregnant women (>20 weeks), 15 mcg *H1N1v* unadjuvanted
- Group D: Non-pregnant mothers, 7.5 mcg *H1N1v* full MF59 adjuvant

The adjuvanted formulations are those used in the *H1N1v* pivotal trials (ongoing). The dose of MF59 refers to the MF59 content of the marketed seasonal influenza vaccine Fluad (marketed in Europe for elderly subjects).

Due to low recruitment rate recruitment to Group B was stopped after 4 months.

Test Vaccines:

- Group A & D: Subunit monovalent influenza vaccine containing 7.5 mcg HA of the H1N1sw flu strain + full MF59. Volume to be injected 0.5mL.
- Group B: Subunit monovalent influenza vaccine containing 3.75 mcg HA of the H1N1sw flu strain + half MF59. Volume to be injected 0.25mL.
- Group C: Subunit monovalent influenza vaccine containing 15 mcg HA of the H1N1sw flu strain without MF59. Volume to be injected 0.5mL.

Amount of antigen and adjuvant in the three formulation and the respective volumes are described in the table below:

Table 1: Antigen content for vaccine formulation

| Group | **Vaccine formulation** | | | **Volume for Injection (mL)** |
| --- | --- | --- | --- | --- |
|  | **Antigen content** | **MF59 content** | |  |
|  | **(in µg)** | **(in mg)** | **(in %)** |  |
| **A & D** | 7.5 | 9.75 | 100 | 0.5 |
| **B** | 3.75 | 4.875 | 50 | 0.25 |
| **C** | 15 | - | 0 | 0.5 |

The study includes sequential blood draws in each enrolled woman to assess persistence of *H1N1v* antibodies following a single vaccination. Blood will be sampled: (I) prior to vaccination; (II) 3 weeks after vaccination; (III) 3 months after vaccination (pregnancy week 36); (IV) 9 months after vaccination (6 months after birth); and (V) 15 months after vaccination (12 months after birth).

SAEs, onset of chronic diseases, Adverse Events of Special Interest (including neuritis, convulsions, severe allergic reactions, angioedema, non-infectious encephalitis, vasculitis, Guillain-Barrè syndrome, demyelination, Bell’s palsy), and AEs that lead to withdrawal from the study and related prescription medications will be collected for the entire study period. The study includes regular follow-up visits of mothers and their infants for safety assessment, up to 12 months after birth.

Cord blood from pregnant women receiving vaccination will be tested for *H1N1v* antibodies to explore the passive immunity transferred to the infant.

This protocol is a nested study within an ongoing prospective, longitudinal unselected birth cohort study of 800 pregnant mothers and their newborn children. The mothers are enrolled during 2^nd^ trimester starting Q1-2009 and until Q4-2010. The study is conducted at a clinical research center in East-Denmark. The first 300 pregnant women have been enrolled in the period before *H1N1v* was circulating broadly in the community and before vaccine became available, and the remaining will be enrolled during the soon to be expected local epidemic.

## 4.2 Sample Size and Power Estimate

This novel cohort enrolls an unselected group of 800 pregnant mothers during 2^nd^ trimester providing the following groups for comparison:

- 400 pregnant women will be randomized to receive *H1N1v* vaccination during pregnancy in one of the three treatment arms with active vaccination. Assuming 25% refusal (18) this will provide approx 100 women in each treatment arm.
- Mothers naïve to *H1N1v* infection or vaccination will be offered vaccination in the fourth study arm, providing a minimum of 100 evaluable subjects.

Based on previous studies of immunity after vaccination(10) the expected sample size of 400 women should be sufficient to ensure adequate estimates for the endpoints specified in the CHMP criteria.

## 4.3 Recruitment strategy

We have established a monthly surveillance in Eastern Denmark of reimbursement to general practitioners for the obligatory pregnancy visit. This allows us to identify all pregnant women during 2^nd^ trimester. We are mailing approx 2.000 invitations per month and receive positive response from approx 10% which are many more than needed for the current protocol.

Detailed study information’s are mailed to the pregnant women. Women who maintain an interest are further informed by telephone and invited for a 2-hour visit at the COPSAC Translational Clinical Research Center for thorough information about the planned study procedures.

Recruitment of pregnant women was initiated early 2009 and the implemented recruitment strategy has successfully recruited according to schedule with 300 currently enrolled as of September 2009. A steady enrollment of 10 families per week will bring the cohort up to the target of 800 by Q4-2010.

This clinical follow-up study is conducted at the COPSAC Translational Clinical Research Center in Copenhagen. To cover a larger geographical region COPSAC is partnering with a satellite research clinic at Naestved Hospital outside Copenhagen. COPSAC has been partnering for many years with this department, the main pediatric dept. in Eastern Denmark Copenhagen. We have established a local research unit working under the auspices of the COPSAC Translational Clinical Research Center. Recruitment at the two centers will be split 3:1.

## 4.4 Participant Selection

### 4.4.2 Inclusion criteria

- Healthy pregnant women living in Eastern Denmark
- Fluent in Danish

### 4.4.2 Exclusion criteria

- Heart disease, endocrine disease, tuberculosis and sarcoidosis.
- History of any anaphylaxis, serious vaccine reactions, or hypersensitivity to influenza viral proteins, to any excipients, and to eggs (including ovalbumin), and chicken proteins

## 4.5 Study Withdrawal

Participants may withdraw from this randomized trial at any time at their request.

## 4.6 Recruitment Bias

The primary aim of the established birth cohort study is to study the immune deregulation from gene-environment interactions occurring during pregnancy and early infancy that may lead to asthma, eczema and allergy. Mothers are enrolled in a double-blind, randomized, controlled trial studying maternal supplementation with marine n-3 polyunsaturated fatty acids (n-3 PUFA) and/or vitamin D on disease development in the infant ([www.clinicaltrials.gov](http://www.clinicaltrials.gov/): Identifier NCT00856947 and NCT00798226). Therefore, the recruitment will likely be biased towards families with increased presentation of asthma, eczema and allergy.

Approximately 90% of the Danish population is of European decent. The requirement for fluent Danish further limits the representation of other races.

## 4.7 Immunogenicity End-Points

The measures for immunogenicity as determined by HI are as follows:

1. Geometric mean HI titer (GMT) on Day 1, Day 22, and at all the other relevant time points to assess persistence;
2. Day 22/Day 1, and at all the other relevant time points to assess persistence/Day 1 geometric mean ratio (GMR) of HI;
3. Percentage of subjects achieving a seroconversion or a significant increase (defined as: HI ≥1:40 for subjects negative at baseline [<1:10]; a minimum 4-fold increase in HI titer for subjects positive at baseline [HI≥1:10]) on Day 22 and at all the other relevant time points to assess persistence;
4. Percentage of subjects with a HI titer ≥1:40 (i.e. seroprotection) on Day 1, Day 22, and at all the other relevant time points to assess persistence.

The same measurements will also be applied to the cord blood samples.

*Criteria for success as determined by HI:*

The immunogenicity criteria for success, as determined by HI, related to the EMEA/CPMP/VEG/4717/2003-Rev.1 (pandemic guideline) and EMEA/CHMP/VWP/263499/2006 (pre-pandemic guideline) are:

For adults subjects aged 18-60 years:

- The percentage of subjects with seroconversion or significant increase in HI antibody is > 40%
- The percentage of subjects achieving an HI titer ≥40 is > 70%
- The GMR is >2.5

The assessment of criteria is based on the corresponding proportions and geometric means ratio empirically found (i.e. on the point estimates).

*Pairwise comparisons:*

Pairwise comparisons between the four groups related to the response after one dose of vaccine (i.e., at Day 22) will be performed in terms of:

- GMTs ratio
- Seroprotection rates differences
- Seroconversion or Significant increase rate differences

Two-sided 95% CI around the point estimates will presented for each endpoint.

## 4.8 Safety Endpoints

The safety of the study vaccines will be assessed based on number of subjects exposed to study vaccines with reported selected adverse events per vaccine group.

Adverse events will include SAEs, onset of chronic diseases, Adverse Events of Special Interest (including neuritis, convulsions, severe allergic reactions, angioedema, non-infectious encephalitis, vasculitis, Guillain-Barrè syndrome, demyelination, Bell’s palsy), AEs that lead to subject’s withdrawal and abmormal events occurring during pregnancy, labor and delivery, and will be collected throughout the whole study period.

The outcomes of pregnancies will be categorized as normal, abnormal or therapeutic/elective termination.

# 5 Clinical Study Plan

## 5.1 Clinical Study Plan, Pregnant Mothers

Pregnant women accepting participation are interviewed on demographics, environmental exposures and personal medical history including medical conditions, smoking history and current medication. Particularly, information will be sought on women’s history of influenza (seasonal and *H1N1v*) and vaccination history and use of any antiviral treatments since 2008.

The women will be requested to monitor *H1N1v* influenza-like illness including that of other household members.

The study includes sequential blood draws in each enrolled woman to assess persistence of *H1N1v* antibodies following a single vaccination. Blood will be sampled:
(I) prior to vaccination
(II) 3 weeks after vaccination
(III) 3 months after vaccination
(IV) 10 months after vaccination

# 6 Work Packages

## WP-1 Cohort Infrastructure

### WP-1.1 Recruitment of 800 pregnant women during 2^nd^ trimester

Recruitment of pregnant women was initiated early 2009 and the implemented recruitment strategy has successfully recruited according to schedule with 300 currently enrolled as of September 2009. A steady enrollment of 10 families per week will bring the cohort up to the target of 800 by Q4-2010.

### WP-1.2 Recruitment of newborns into the ABC birth cohort

The newborns are enrolled at a visit to the research unit one week after birth, which includes detailed history of family heredity, demographics, socio-economics and exposures during pregnancy and in the home environment. A thorough physical examination includes anthropometrics and sampling for microbiology surveillance.

### WP-1.3 Database for on-line data capture

A dedicated standard query language (SQL) database for on-line collection of clinical data has been developed specifically for this birth cohort study. This database will be adapted to the current protocol and allows real-time monitoring of the status of the cohort.

### WP-1.4 Data monitoring

This clinical study is monitored in accordance with "Good Clinical Practice" by the Copenhagen University GCP Unit.

## WP-2 Clinical surveillance

### WP-2.1 Scheduled visits

Pregnant women are scheduled for clinic visits after week 20 for study enrollment, blood sampling and vaccination in accordance with the single-blind, randomized treatment schedule. The women will return for sequential blood draws to assess persistence of *H1N1v* antibodies after 3 weeks and 3 and 10 months. Safety follow up will last for 12 months after birth for both the mother and the newborn.

### WP-2.4 SAE reporting

All SAEs which occur during the course of the trial, whether considered to be associated with the study vaccination or not, have to be reported within 24 hours or at the latest on the following working day by telephone or fax to either of the following:

Study Sponsor: Copenhagen Studies on Asthma in Childhood; Danish Pediatric Asthma Center; Health Sciences, University of Copenhagen; University Hospital, Gentofte, Phone +45 39777360; Fax +45 39777129.

For trial related emergencies out of office hours please contact mobile phone +45 39777382

Principal Investigator: Anne Louise Bischoff , MD, PhD student

Novartis Medical Contact: Dr. Volker Brauer, Novartis Vaccines & Diagnostics, Marburg, Germany, FAX: +49 6421 39 2826

As far as possible, all points raised on the “Serious Adverse Event” form need to be addressed and faxed immediately to the Study Monitor. The original must be retained by the investigator. The event must also be documented on the Adverse Events CRF. After receipt of the initial report, the Study Monitor/Sponsor will review the information and contact the investigator if it is necessary to obtain further information for assessment of the event. Any medication or other therapeutic measures used to treat the event will be recorded on the appropriate CRF(s) in addition to the outcome of the AE. Any serious adverse reaction must be reported to the EC in a timely manner, according to local regulations. Adequate documentation will be provided to the sponsor and Novartis Vaccines showing that the EC has been properly notified. The sponsor must also comply with the applicable regulatory requirement(s) related to the reporting of unexpected serious and non-serious adverse drug reactions to the local EC. Novartis, as provider of the clinical vaccine will be responsible for reporting cases to regulatory authorities, as applicable, copying the sponsor on all these reports.

All adverse events that are considered as severe and, at the same time, at least possibly related to the Study Vaccine should be reported on a monthly basis for the whole duration of the study to Dr Volker Brauer FAX: +49 6421 39 2826 at Novartis V&D Marburg, Germany.

The sponsors and investigators responsibilities as regards reporting SAEs and SUSARs in accordance with the European Directive 2001/20/EC will be transferred to Novartis Vaccines.

If required, a follow up report including all new information obtained on the SAE must be prepared and sent to the Study Monitor, or it will be collected by a representative of the sponsor. The report should be marked “Follow-up report.”

The investigator will submit, on request, copies of all these reports to the EC and other relevant authorities.

*Post-Study Events*

Any AE occurring at any time outside the observation period or after the end of the study and considered to be caused by the study vaccine - and therefore a possible adverse drug reaction - must be reported to the sponsor and to Novartis Vaccines.

## WP-3 H1N1v analyses

Serum samples will be assessed by the means of strain-specific hemagglutination inhibition (HI) assay.

Heterologous influenza strain HI testing, as well as additional assays (e.g. MN and / or SRH) to better characterize immune response might be performed as well.

HI assay will be performed at Novartis Vaccines, Clinical Serology Laboratory, Marburg, Germany, or a delegate laboratory.

## WP-4 Vaccine Supply, Storage, Tracking and Labeling

Novartis Vaccines will supply the investigational vaccine. The investigator should acknowledge receipt of the investigational study vaccines. Study vaccines must be received by a designated person at the study site, handled and stored safely and properly, and kept in a secured location to which only the investigator or designee have access. Upon receipt, investigator or designee should ensure study vaccines are received in good condition. The vaccines at the site must not be used before the appropriate shipping conditions have been checked and confirmed by Sponsor staff. Study vaccine will be labeled and will comply with the legal requirements of the Country. All study vaccines should be stored according to the instructions specified on the labels.

Vaccines that have been stored differently from the Novartis Vaccines recommendations **must not** be used unless Novartis Vaccines provides written authorization for use. In the event that the use cannot be authorized, vaccine supply must be replaced with fresh stock supplied by Novartis Vaccines. Batches of commercially available comparator vaccines must be stored separately from normal hospital/practice stocks.

The investigator should ensure that the investigational product(s) delivered to the site is used only in accordance with the approved protocol. Monitoring of vaccine accountability will be performed by the study monitor during site visits and at the completion of the trial.

The investigator should maintain an accurate record of products delivery to the site, the inventory at the site, the administration to the subjects, and the return to Novartis Vaccines of unused study vaccines. These records should include dates, quantities, batch number, expiration dates and the unique identifying number assigned to the investigational product.

A detachable label will be found either on the outer box or on the label of the primary container of the investigational product. The detachable label contains a unique identifier for each dose. The investigator must stick the detachable part on the administration log upon dispensing of the vaccine to certify that the vaccine was effectively administered and for tracking purposes.

At the conclusion of the study, and as appropriate during the course of the study, the investigator should return all unused study vaccines, packaging and supplementary labels to Novartis Vaccines and Diagnostics.

If the unused study vaccines are disposed at the site, the investigator should provide a copy of the site’s procedure for destruction of hazardous material and documentation of the destruction. For the information that must be included in the documentation of destruction please refer to related Novartis Vaccines SOP.

## WP-5 Processing, Labeling and Storage of Serum Samples for Serology

Approx. 15mL of blood sample should be drawn in order to obtain the minimum required serum volume for immunological assays. Blood will be sampled: (I) prior to vaccination (pregnancy week 24); (II) 3 weeks after vaccination (pregnancy week 27); (III) 3 months after vaccination (pregnancy week 36); (IV) 9 months after vaccination (6 months after birth); and (V) 15 months after vaccination (12 months after birth).

Blood taken for immunological assessment will be centrifuged preferably on the same day and the serum will be equally distributed in 2 cryo tubes provided giving an “original” and a “duplicate” samples. “Original” and “duplicate” aliquots will be stored in separate storage boxes at a temperature of minus 18°C or below.

For the labeling a set of serum tube and shipping log labels will be provided to the Investigator containing a uniquely identifying barcode with the following information preprinted: 1) protocol number; 2) the visit number; 3) the subject number. The subject code requires to be added to the label by handwriting. The serum tube labels are placed lengthwise on the tube prior to the serum separation.

For each shipment, the shipping log, an inventory of the samples which accompanies the shipment, must be prepared. The information requested on the shipping log must be completed by a responsible person at the site shortly before the shipment is initiated. A copy of the shipping log has to be placed into the investigators study file.

Detailed instructions for labeling, storage and shipping of serum samples are included in the Serology Guideline, provided separately.

# 7 Data Management

## 7.1 Data Collection

All study data are being collected on-line during the clinic visits into a dedicated Standard Query Language Database on a Novel server. Symptom recordings from diary cards are reviewed with the mother by the doctor at the research unit and entered into the database, where they are locked after double-check. The database was built for the ongoing COPSAC cohort study and has been further developed to this novel birth cohort with specific adjustments to capture the data for the current protocol. Objective measurements are double-checked. An audit trail in the data-base monitors any changes. The database is locked after data-check.

## 7.2 Record Retention

All records are kept confidential. All documents listed in the ICH Good Clinical Practice (GCP) Guideline will be retained in a locked and secure storage facility for a minimum of fifteen years, per DHHS (45 CFR 46.115(b)).

## 7.3 Data Monitoring

This ongoing birth cohort study is conducted in accordance with the requirements of GCP as defined in Guidelines, EU Clinical Trials Directive (2001/20/EC), and EU GCP Directive (2005/28/EC). All study participants must sign all applicable approved informed consent forms prior to any study-related procedures. The confidentiality of all study participants will be protected in accordance with GCP Guidelines.

The study is being monitored by the Copenhagen University GCP-unit in accordance with these guidelines.

## 7.4 Data Analysis Plan

### Primary end-point analysis

The *H1N1v*-specific antibodies measured longitudinally during pregnancy will be analyzed immediately upon completion of the last pregnancy included.

The *H1N1v*-specific antibodies measured longitudinally after pregnancy will be analyzed immediately upon completion of the last follow-up visit.

*Secondary end-point analysis*

The *H1N1v*-specific antibodies in cord blood will be analyzed as an exploratory end-point.

The frequency and severity of infant lower respiratory illnesses will be compared by the vaccination status of their mothers.

## 7.5 Data Reporting

The on-line recording of all data allows real-time reporting of the study outcome. Quarterly summary reports will be provided to the public and at our website [www.copsac.com](http://www.copsac.com/)

## 7.6 Data Sharing

Health authorities and sponsors will have full access to data. Publication of such data will conform to the Vancouver Guidelines on scientific merit for publication.

# 8 Legal and Ethical Aspects

The proposed project is based on a clinical study involving pregnant women and therefore a strong focus on ethical and legal aspects is mandatory. The main birth cohort study has been approved by the Danish Ethical Committee (H-B-2008-093) and the Danish Data protection agency (2008-41-2599). The current protocol extension has been submitted for approval at both agencies.

The proposed project will focus on elucidating vaccine efficiency during the 2^nd^ and 3d trimester as there is little documented experience with adjuvanted vaccines used in 1^st^ trimester of pregnancy.

There are not expected to be any severe risk associated with the present study based on experiences with previous vaccines and results from Phase 1 and 2 trials. Routine vaccination of pregnant women is considered safe and recommended in several countries including the USA.

The individual risk of severe disease from influenza is small. The potentially increased risk associated with decreased protection in the study arms with less or no adjuvant will therefore be small in absolute terms and ethically justifiable.

Preliminary trials using the MF59 adjuvanted vaccine reported mild to moderate reactions towards vaccination. The study showed that the most frequent local and systemic reactions were pain at the injection site and muscle aches which were noted in 70% and 42% of the subjects respectively.(9) In addition MF59 has been approved and successfully used in Europe for over a decade as the adjuvant in *Fluad* administered to elderly people not capable of mounting a sufficient immune response following immunization.

COPSAC Translational Clinical Research Center is highly experienced in conducting clinical research in pregnant women and infants through the previous COPSAC birth cohort study and a long track-record of randomized controlled trials as reflected in a number of key-publications.(11-17;19)

Two nested studies in the original COPSAC cohort evaluated the ethical aspects of performing such comprehensive and invasive clinical study in early childhood and found that clinical studies can be performed in a manner that is perceived ethically sound by parents(20) and is well accepted by the children.(21) These experiences from the COPSAC cohort study will be transferred to the current project.

The mission of the COPSAC Translational Clinical Research Center is to promote evidence-based health management of children through research. It is only through research that we can assure lasting improvements of the welfare of children through an evidence-based health service. We therefore have an ethical obligation to perform high-quality clinical studies like the proposed in order to provide the evidence base for health recommendations, policies and treatment with potential health effects for millions of individuals.

# 9 Study Impact

## 9.1 Innovative value

The innovative value of this proposed research alliance is partly due to this “experiment by nature” whereby the pandemic expected from Q4-2009 will divide our ongoing birth cohort study into two groups who will be exposed vs. never-exposed;

The current protocol draws upon the logistics and the wealth of basic scientific data harvested within the main protocol including genome-wide association analysis and longitudinal assessments of the immune profile and the microbiology and exposure assessments. The prominent translational medicine approach with close collaboration between clinical research and basic science will provide novel insight into both the optimal vaccination strategy for *H1N1v* during pregnancy.

## 9.2 Publication and promotional strategy

Real-time reporting from the observed *H1N1v*-cases will be presented on our website [www.copsac.com](http://www.copsac.com/)

Summary reports on the study progress will be provided on a quarterly basis.

The study design including the current proposal will be published in an international peer-reviewed journal to provide other researchers with information on the ongoing project and support an international collaborative effort.

Research data will be disseminated through publication in high impact scientific journals. Also broadly oriented presentation will be assured through secondary publication in non-professional health related media. The high public interest in this topic guarantees high public media coverage following publication of results. Every effort will be made assuring that the implication of study results are presented in a balanced manner and are not over-interpreted as is often the case when individual study results on health related subjects are presented in public media.

# 10 Feasibility and Project Management

## 10.1 Research Management:

COPSAC Translational Clinical Research Center is an international centre of excellence in peri-natal and pediatric research and structured as an independent institution affiliated to the University of Copenhagen and the University Hospital, Gentofte. The core competence of the center is the clinical phenotyping in early life. It is headed by professor Hans Bisgaard with 20 employees including an academic staff of 14 all working towards the common goal of translational research in the COPSAC longitudinal birth cohort: [www.copsac.com](http://www.copsac.com/) Professor Hans Bisgaard, Health Sciences, University of Copenhagen will be the daily responsible coordinator of all activities. He is founder and head of the COPSAC Translational Clinical Research Center as well as the Danish Pediatric Asthma Center. The management will be integrated into the existing administration structure at the COPSAC Translational Clinical Research Center at Gentofte hospital, Copenhagen, with its business manager, data manager, project coordinator and finance controller.

| **Figure 4: Management Matrix** | | | |
| --- | --- | --- | --- |
| **Partners**  /Steering committee | **Senior Researchers** | **PhD students** | **Research Assist.** |
| Prof. H Bisgaard, MD, DMSci  COPSAC Translational Clinical Research Center | K Bønnelykke, MD  P Sleiman, MSc  A Mølgaard, PhD  TR Jørgensen, PhD  G Palludan-Müller, MSc  C Bang Pedersen, MSc | B Chawes, MD  C Giwercman, MD  AL Bischoff, MD  N Følsgaard, MD  J Stokholm, MD  L Pedersen, MD | L Vind  K Hinsby  L Klansø  M Holmberg  H Wellemberg  L Toft-Jensen  AJ Pedersen |

## 10.2 Governance

We have established a robust and transparent governance structure for our birth cohort studies with a consistent management, cohesive policies, processes and decision-rights.

The coordinator role will be managed by the Principal Investigator who will organize reporting and dissemination of activities.

A steering committee for this protocol will be formed by the principal investigator and the co-investigators. The steering committee will meet every 3 months to evaluate the program, decide if adjustments to the project are necessary, assure that the project is performing as scheduled, suggest contingency planning when needed, and take responsibility that the project activities are consistent with the overall objectives. The steering committee will have decisive power over the project.

# 11 Co-Funding

The Center is funded by private and public research funds. The core clinical research center is partly funded by the State budget.

The present proposal is only possible within the proposed budget and timeframe because the cohort was initiated early 2009. This initial funding was provided as part of a Translational Research Center funding by the Lundbeck Foundation donated after international competition to the COPSAC Translational Clinical Research Center. This has allowed funding of the recruitment of the cohort and short-term follow up with a narrow focus on the association between airway bacterial colonization and asthma discovered in the COPSAC cohort.

The current protocol is entirely dependent on new complementary funding.

# 13 References

(1) Jamieson DJ, Honein MA, Rasmussen SA, Williams JL, Swerdlow DL, Biggerstaff MS et al. H1N1 2009 influenza virus infection during pregnancy in the USA. Lancet 2009 August 8;374(9688):451-8.

(2) Baker MG, Wilson N, Huang QS, Paine S, Lopez L, Bandaranayake D et al. Pandemic influenza A(H1N1)v in New Zealand: the experience from April to August 2009. Euro Surveill 2009;14(34).

(3) Dawood FS, Jain S, Finelli L, Shaw MW, Lindstrom S, Garten RJ et al. Emergence of a novel swine-origin influenza A (H1N1) virus in humans. N Engl J Med 2009 June 18;360(25):2605-15.

(4) Chowell G, Bertozzi SM, Colchero MA, Lopez-Gatell H, puche-Aranda C, Hernandez M et al. Severe respiratory disease concurrent with the circulation of H1N1 influenza. N Engl J Med 2009 August 13;361(7):674-9.

(5) Bhat N, Wright JG, Broder KR, Murray EL, Greenberg ME, Glover MJ et al. Influenza-associated deaths among children in the United States, 2003-2004. N Engl J Med 2005 December 15;353(24):2559-67.

(6) Keren R, Zaoutis TE, Bridges CB, Herrera G, Watson BM, Wheeler AB et al. Neurological and neuromuscular disease as a risk factor for respiratory failure in children hospitalized with influenza infection. JAMA 2005 November 2;294(17):2188-94.

(7) Coffin SE, Zaoutis TE, Rosenquist AB, Heydon K, Herrera G, Bridges CB et al. Incidence, complications, and risk factors for prolonged stay in children hospitalized with community-acquired influenza. Pediatrics 2007 April;119(4):740-8.

(8) Zaman K, Roy E, Arifeen SE, Rahman M, Raqib R, Wilson E et al. Effectiveness of maternal influenza immunization in mothers and infants. N Engl J Med 2008 October 9;359(15):1555-64.

(9) Clark TW, Pareek M, Hoschler K, Dillon H, Nicholson KG, Groth N et al. Trial of Influenza A (H1N1) 2009 Monovalent MF59-Adjuvanted Vaccine -- Preliminary Report. N Engl J Med 2009 September 10.

(10) Greenberg ME, Lai MH, Hartel GF, Wichems CH, Gittleson C, Bennet J et al. Response after One Dose of a Monovalent Influenza A (H1N1) 2009 Vaccine -- Preliminary Report. N Engl J Med 2009 September 10.

(11) Bisgaard H, Hermansen MN, Loland L, Halkjaer LB, Buchvald F. Intermittent inhaled corticosteroids in infants with episodic wheezing. N Engl J Med 2006 May 11;354(19):1998-2005.

(12) Bisgaard H, Hermansen MN, Buchvald F, Loland L, Halkjaer LB, Bonnelykke K et al. Childhood asthma after bacterial colonization of the airway in neonates. N Engl J Med 2007 October 11;357(15):1487-95.

(13) Sleiman PMA, Flory J, Imielinski M, Bradfield JP, Annaiah K, Willis-Owen SAG et al. Common variants in DENND1B associate with pediatric asthma. N Engl J Med 2009;in press.

(14) Bisgaard H, Loland L, Holst KK, Pipper CB. Prenatal determinants of neonatal lung function in high-risk newborns. J Allergy Clin Immunol 2009 January 17.

(15) Andersen ZJ, Loft S, Ketzel M, Stage M, Scheike T, Mette MN et al. Ambient Air Pollution Triggers Wheezing Symptoms in Infants. Thorax 2008 February 11.

(16) Bisgaard H, Simpson A, Palmer CN, Bonnelykke K, McLean I, Mukhopadhyay S et al. Gene-environment interaction in the onset of eczema in infancy: filaggrin loss-of-function mutations enhanced by neonatal cat exposure. PLoS Med 2008 June 24;5(6):e131.

(17) Bisgaard H, Bonnelykke K, Sleiman PM, Brasholt M, Chawes B, Kreiner-Moller E et al. ORMDL3 Associated Gene Variants are Associated with Asthma and Exacerbations but not Atopy in Early Childhood. Am J Respir Crit Care Med 2008 November 21.

(18) Kmietowicz Z. Opposition to swine flu vaccine seems to be growing worldwide. BMJ 2009;339:b3461.

(19) Palmer CN, Irvine AD, Terron-Kwiatkowski A, Zhao Y, Liao H, Lee SP et al. Common loss-of-function variants of the epidermal barrier protein filaggrin are a major predisposing factor for atopic dermatitis. Nat Genet 2006 April;38(4):441-6.

(20) Gammelgaard A, Knudsen LE, Bisgaard H. Perceptions of parents on the participation of their infants in clinical research. Arch Dis Child 2006 December;91(12):977-80.

(21) Gammelgaard A, Bisgaard H. Seven-year-old children's perceptions of participating in a comprehensive clinical birth cohort study. Clinical Ethics 2009;4:79-84.
